# Fungal diversity of mangrove-associated sponges from New Washington, Aklan, Philippines

**DOI:** 10.1080/21501203.2018.1518934

**Published:** 2018-09-17

**Authors:** Mark S. Calabon, Resurreccion B. Sadaba, Wilfredo L. Campos

**Affiliations:** a National Institute of Molecular Biology and Biotechnology, University of the Philippines Visayas, Miagao, Philippines; b Division of Biological Sciences, College of Arts and Sciences, University of the Philippines Visayas, Miagao, Iloilo, Philippines; c OceanBio and MarineBio Laboratories, Division of Biological Sciences, College of Arts and Sciences, University of the Philippines Visayas, Miagao, Iloilo, Philippines

**Keywords:** Host-preference, mangrove sponges, marine fungi, sponge-associated fungi, tropical mycology

## Abstract

Sponge-associated fungi are the least explored marine fungal groups. It is only in recent years that fungal symbionts of marine sponges have received attention mainly due to the isolation of bioactive metabolites while not much attention was given to their specificity, biogeography and exact roles in marine sponges. The diversity of fungi associated with mangrove sponges (*Axinella* sp., *Halichondria* cf. *panicea, Haliclona* sp., *Tedania* sp.) collected from New Washington, Aklan, Philippines were investigated using morphological observation. A total of 110 species of sponge-associated fungi belonging to 22 genera of ascomycetes with 18 genera of asexual morphs whose sexual stage is unknown, 2 genera of basidiomycetes, 21 morphospecies of *Mycelia sterilia*, 1 unidentified yeast species and 11 unidentified hyphomycetes were isolated from four species of mangrove sponges. This is the first study that explored the diversity and ecology of sponge-associated fungi in mangrove habitats from the Philippines. The results of the study suggest host-preference by various fungal taxa and the development of fungi on these hosts appeared to be strongly influenced by the characteristics or nature of the immediate environment.

## Introduction

Fungi have long been known to exist in the marine environment but considered to be the underexplored group in the oceans compared to bacteria, plants and animals (Jones and Pang 2012). Marine fungi live as saprophytes, parasites and symbionts on various matrices such as sediments, logs, water, as well as algae, vascular plants, invertebrates and fishes (Johnson and Sparrow 1961; Kohlmeyer and Kohlmeyer 1979). Marine fungi can be classified as true or obligate and facultative marine fungi. The former can complete their lifecycle in marine environments and the latter can grow in freshwater or terrestrial habitats as well as in marine environments (Kohlmeyer and Kohlmeyer 1979). However, Jones et al. (2015) accept a wider interpretation of what can be considered marine.

Sponges are known inhabitants of coastal and deep-sea environments. They are filter feeders and known to harbour diverse groups of microbes (Hentschel et al. 2006; Taylor et al. 2007). Marine sponges are a rich source of compounds with bioactive potential. A review by Blunt et al. (2012) reported that 296 new compounds from marine sponges were isolated only in 2011. But over the past decade, a consensus has developed among experts that these novel natural products from sponge extracts are synthesised, either in part or in entirely, by the symbiotic microbes that are intimately associated with these marine metazoans (Konig et al. 2006; Meenupriya and Thangaraj 2010). These microbes are thought to be involved in a variety of ecological functions including production of secondary metabolites that can contribute to their own ecological success and to that of their host (Höller et al. 2000). The previous work on marine microbial diversity in sponges focuses mostly on bacterial associates with proposed novel phylum candidate *Poribacteria* (Fieseler et al. 2004; Kamke et al. 2014). It is only in recent years that the fungal symbionts of marine sponges have received attention (Gao et al. 2008; Wang et al. 2008; Baker et al. 2009; Li and Wang 2009; Menezes et al. 2010; Debbab et al. 2011) mainly related to their capacity to produce novel bioactive metabolites (Proksch et al. 2003; Bugni and Ireland 2004; Amagata et al. 2006; Blunt et al. 2009; Aly et al. 2011; Jones 2011).

Fungi have been isolated from subtidal sponges in tropical, subtropical and temperate countries. (Höller et al. 2000; Wang et al. 2008; Caballero-George et al. 2010; Paz et al. 2010; Zhou et al. 2011; Thirunavukkarasu et al. 2012; Flemer 2013; Henriquez et al. 2013; Bolaños et al. 2015). In Asia, there have been published information on the diversity of sponge-associated fungi in countries like China (Zhang et al. 2009; Liu et al. 2010; Ding et al. 2011; Zhou et al. 2011; Yu et al. 2012; He et al. 2014; Jin et al. 2014), India (Meenupriya and Thangaraj 2010; Thirunavukkarasu et al. 2012) Indonesia (Namikoshi et al. 2002), Israel (Paz et al. 2010), Malaysia (Mahyudin 2008) and Russia (Pivkin et al. 2006). To date, there are no ecological studies of sponge-associated fungi or any information about the species richness and fungal biodiversity of sponges in the marine environment of the Philippines. Furthermore, there is no published information about the fungal diversity of mangrove-associated sponges. The present study aims to determine the diversity of fungi associated with different species of mangrove-associated sponges collected from the mangrove area of New Washington, Aklan, Philippines. The assessment of fungal diversity will be of great value to understand the fungal ecology in mangrove-associated sponges and to explore the biotechnological potentials of these fungi. The additional information obtained from this study will serve as baseline information on the fungal composition of the different sponge species in the Philippines.

## Materials and methods

### Study site

**Figure 1.**
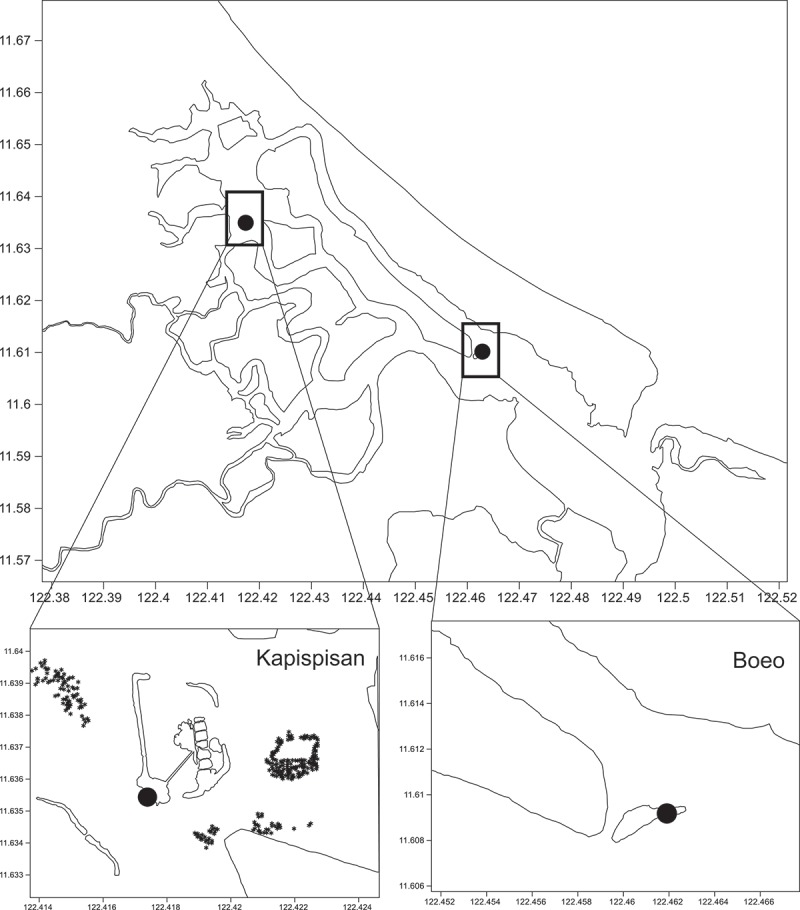
Map showing two sampling sites in New Washington, Aklan, Philippines (red dot: Bo-eo, Pinamuk-an, New Washington, Aklan; yellow dot: Kapispisan, Pinamuk-an, New Washington, Aklan).

New Washington, Aklan is located on the north-eastern part of Panay Island. It is a coastal community composed of islets surrounded by brackish water rivers that forms part of the Batan Estuary and the northern coast of the island faces the Sibuyan Sea. Two sites, Kapispisan (11° 38ʹ 5.748” N, 122° 25ʹ2.388” E) and Boeo (11° 36ʹ 33.804 N, 122° 27ʹ 42.119 E), both in Pinamuk-an, New Washington, Aklan, were surveyed for mangrove sponges by mask and snorkel to a depth of 1–2 m in September 2015 (Figure 1). The mangrove species that dominated Pinamuk-an River include *Sonneratia alba, Avicennia umphiana* and *Avicennia marina* (Ochavo et al. unpublished).

### Collection and identification of mangrove-associated sponges

Sponges (*Axinella* sp., *Halichondria* cf. *panicea, Haliclona* sp., *Tedania* sp.) attached to the mangrove roots were collected and photographed for identification purposes. Latex glove was worn for the collection of sponges and samples were removed from the mangrove roots using scalpel or by directly cutting the roots with attached sponge. Sponge samples (~20–25 g) were transferred directly into zip-lock bags containing seawater from the collection sites to prevent direct contact with air. Sponges were stored and transported immediately in a cooler box back to the laboratory for processing within 24 h to avoid external microbial contamination and excessive proliferation of the samples. All the epiphytic faunas attached to the samples were removed.

### Isolation of fungi

Sponge-associated fungi were isolated in the Microbiology Laboratory of University of the Philippines Visayas – National Institute of Molecular Biology and Biotechnology (UPV – NIMBB). Sponge tissue segments were rinsed three times with sterile artificial seawater to eliminate adherent surface debris and contaminants (Thirunavukkarasu et al. 2012). The inner tissue (middle internal mesohyl area) was excised with a sterile scalpel and was cut into small pieces. Ten grams of each sample was homogenised using a blender containing 20 ml sterile artificial seawater under aseptic conditions. One millilitre of the resulting homogenate was transferred to a sterile test tube containing 9 ml of 0.85% NaCl and mixed in the vortex (dilution 10^−1^). The serial dilution was repeated until 10^−5^ has been reached.

For fungal cultivation, 100 µl of dilutions 10^−0^ to 10^−5^ were plated in triplicate onto 15 ml of the selected isolation media supplemented with penicillin G and streptomycin (100 µg/ml each) using spread plate technique. All five media (Czapek Dox Agar, Cornmeal Agar, Mycobiotic Agar, Potato Dextrose Agar, Rose Bengal Agar) were added with 1.5% NaCl which is the salt concentration used in isolation of mangrove fungi in the Philippines. A control plate was prepared by exposing a blank plate in the middle of the working area for 15 min. The inoculated plates were sealed with Parafilm^TM^ and incubated at room temperature (27°C) in inverted position and examined daily for the appearance of fungal colonies up to three weeks, depending on the growth of species. Edges from emerging fungal colonies growing out on culture medium were picked and transferred with a sterile inoculating loop onto culture tube containing fresh media supplemented with antibiotic solution. Mycelia or spores were transferred again in new culture media for purification. For yeasts, cells were streaked onto a fresh culture media. The resulting plates were incubated at room temperature (27°C) for pure culture and purification was done rigorously until a homogenous fungal isolate was obtained for identification.

### Fungal density

The number of colony forming units (CFU) per gram of dry weight of sponge (CFU/g dw) was estimated for each identified fungal species.

### Identification of fungal isolates

Filamentous fungi were identified based on their macroscopic (colonial) and microscopic characteristics. Colonial descriptions included colony characteristics such as colour (reverse and obverse), texture, margin, elevation and characteristics of aerial hyphae. Slide culture technique of Riddell (1950) was employed for microscopic examination of fungal isolates that included spore morphology, colour, shape, wall ornamentation or texture, size, conidial formation and other relevant characteristics such as phialide and conidiation pattern. In the Mycology Laboratory of UPV- Freshwater Aquaculture Station, microscopic examination of colony colours and growth rates was assessed with a dissecting microscope. Microscopic characteristics of fungal isolates were determined by viewing slides with distilled water using a light microscope under Low Power Objective (LPO) and High Power Objective (HPO). Microphotographs of the reproductive structures were taken for identification purposes. Filamentous fungi were identified to at least genus level based on the identification scheme by Kohlmeyer (1984), Kohlmeyer and Volkmann-Kohlmeyer (1991), Barnett and Hunter 1998), Howard (2003), Watanabe (2010), Domsch et al. (2007), Pitt and Hocking (2009) and Campbell et al. (2013) in addition to other available keys and monographs. Identification of yeast isolates was based on keys by Kurtzman and Fell (1998) that included colonial and microscopic characteristics and by using API 20C Aux (bioMerieux, Rome, Italy) that was based on 19 carbohydrate assimilation tests with negative control. *Mycelia sterilia* or fungi that failed to grow and sporulate were given codes using cultural characteristics (e.g. colony surface, texture and hyphal pigmentation). The fungal descriptions in MycoBank (www.mycobank.com) were used as guide for further identification of the fungal isolates.

### Preservation of fungal cultures

Continuous growth method was used for fungal culture preservation. After identification, all pure cultures of fungal isolates were grown on agar slant in a culture tube and stored at 5°C. Fungal cultures were maintained and preserved in National Institute of Molecular Biology and Biotechnology, University of the Philippines Visayas (UPV), Miagao, Iloilo and Mycology Laboratory of the Division of Biological Sciences, College of Arts and Sciences, UP Visayas.

### Data analyses

- Total frequency of occurrence (FOC, %) of fungi in sponge samples was computed using the formula:
  Total FOC (%): number of presence/total sponges *100
  Frequency of occurrence (FOC) of species A (%) per sponge species:

$$\frac{No\;\;.of\;\;collections\;\;of\;\;species\;\;A}{Number\;\;of\;\;samples\;\;examined}\;x\;100$$

Based on the frequency of occurrence, the following groupings were made (Hyde 1989; Hyde and Sarma 2000; Sarma and Raghukumar 2013): very frequent (≥10%), frequent (5–10%), infrequent (1–5%), rare (≤1%).

(B) The diversity of fungi associated with the mangrove sponges was calculated following Ludwig and Reynolds (1988).
  (a) Shannon Index $\left(H^{\prime }\right)=-\sum \left(pi\;ln\;pi\right)$

where: *p_i_* is the proportion of individuals that species *i* contribute to the total number of individuals as shown in the formula below:

pi=ni/N

where: N = total number of individuals (records)

n*i* = number of individuals *i1, i*2, *i*3, *i*4, … *i*x

(b) Simpson Index of Dominance (D)

D=Σpi2

where: *P_i_ = *proportion of individuals in the *i*th species

As *D* increases, diversity decreases. Simpson’s index is usually expressed as:

1−Dor 1/D

(c) Shannon Evenness (*J’*)

$$J^{\prime }=\frac{H^{\prime }}{H^{\prime }max}$$

where: *H’_max_* = maximum value of diversity for the number of species present

(d) Simpson’s Evenness (*E_1/D_*)

$$E1/D=\left(\left(1/D\right)\right)/S$$

W3here: *D = *Simpson’ index of diversity

*S* **= **Species richness

- Jaccard Index of Species Similarity was calculated pair-wise among the hosts based on the presence or absence of each fungal species using the formula

$$JI=a/\left(a+b+c\right)$$

where: *a* is the number of fungal species occurring in both hosts

*b* is the number of fungal species unique to the first host and

*c* is the number of fungal species unique to the second host.

## Results

### Overall fungal populations and diversity

A total of 110 species of sponge-associated fungi belonging to 22 genera of ascomycetes with 18 genera of asexual morphs whose sexual stage is unknown, 2 genera of basidiomycetes, 21 morphospecies of *Mycelia sterilia*, 1 unidentified yeast species and 11 unidentified hyphomycetes were isolated from four species of mangrove-attached sponges collected from New Washington, Aklan, Philippines (Table 1).

**Table 1.** Overall frequency of occurrence (%) of sponge-associated fungi collected from a mangrove area in New Washington, Aklan, Philippines.

		*Halichondria* cf. *panicea*		*Axinella* sp.		*Tedania* sp.		*Haliclona* sp.			
No.	Species	No. of occurrences	FOC (%)	No. of occurrences	FOC (%)	No. of occurrences	FOC (%)	No. of occurrences	FOC (%)	Total no. of occurrences	Total FOC (%)
1	*Acremonium kiliense*	–	–	–	–	–	–	3.00	4.00	3.00	0.95
2	*Acremonium* sp. 1	2.00	2.67	–	–	–	–	–	–	2.00	0.63
3	*Acremonium* sp. 2	2.00	2.67	–	–	–	–	–	–	2.00	0.63
4	*Acremonium strictum*	1.00	1.33	–	–	–	–	–	–	1.00	0.32
5	*Acrodontium* cf. *crateriforme*	29.00	38.67	4.00	5.33	1.00	1.33	2.00	2.67	36.00	11.39
6	*Aspergillus candidus*	–	–	1.00	1.33	–	–	–	–	1.00	0.32
7	*Aspergillus* cf. *fumisynnematus*	2.00	2.67	2.00	2.67	–	–	–	–	4.00	1.27
8	*Aspergillus niger*	13.00	17.33	3.00	4.00	6.00	8.00	4.00	5.33	26.00	8.23
9	*Aspergillus niveus*	1.00	1.33	–	–	–	–	1.00	1.33	2.00	0.63
10	*Aspergillus* cf. *novofumigatus*	–	–	–	–	–	–	1.00	1.33	1.00	0.32
11	*Aspergillus ochraceus*	1.00	1.33	–	–	–	–	–	–	1.00	0.32
12	*Aspergillus* cf. *penicilloides*	–	–	1.00	1.33	–	–	–	–	1.00	0.32
13	*Aspergillus restrictus*	–	–	1.00	1.33	–	–	–	–	1.00	0.32
14	*Aspergillus sclerotiorum*	–	–	1.00	1.33	–	–	–	–	1.00	0.32
15	*Aspergillus* sp. 1	1.00	1.33	–	–	–	–	–	–	1.00	0.32
16	*Aspergillus* sp. 2	–	–	–	–	–	–	1.00	1.33	1.00	0.32
17	*Aspergillus* sp. 3	1.00	1.33	–	–	–	–	–	–	1.00	0.32
18	*Aspergillus* sp. 4	–	–	–	–	1.00	1.33	–	–	1.00	0.32
19	*Aspergillus* sp. 5	1.00	1.33	–	–	–	–	–	–	1.00	0.32
20	*Aspergillus* sp. 6, Sect. Niger	1.00	1.33	–	–	–	–	–	–	1.00	0.32
21	*Aspergillus* sp. 7, Sect. Restrictus	–	–	–	–	–	–	1.00	1.33	1.00	0.32
22	*Aspergillus* sp. 8, Sect. Fumigatus	–	–	2.00	2.67	–	–	–	–	2.00	0.63
23	*Aspergillus* sp. 9, Sect Fumigatus	–	–	1.00	1.33	–	–	–	–	1.00	0.32
24	*Aspergillus* sp. 10, Sect. Flavus	–	–	–	–	–	–	1.00	1.33	1.00	0.32
25	*Aspergillus* sp. 11, Sect. Terreus	1.00	1.33	3.00	4.00	–	–	–	–	4.00	1.27
26	*Aspergillus sydowii*	7.00	9.33	2.00	2.67	2.00	2.67	1.00	1.33	12.00	3.80
27	*Aspergillus tamarii*	1.00	1.33	–	–	–	–	–	–	1.00	0.32
28	*Aspergillus terreus*	1.00	1.33	–	–	1.00	1.33	–	–	2.00	0.63
29	*Beauveria* sp.	–	–	–	–	1.00	1.33	–	–	1.00	0.32
30	*Candida famata*	1.00	1.33	–	–	–	–	–	–	1.00	0.32
31	*Candida guilliermondii*	2.00	2.67	1.00	1.33	1.00	1.33	1.00	1.33	5.00	1.58
32	*Cladosporium cladosporioides*	2.00	2.67	–	–	–	–	1.00	1.33	3.00	0.95
33	*Cladosporium* sp.1	1.00	1.33	2.00	2.67	–	–	–	–	3.00	0.95
34	*Cladosporium* sp. 2	2.00	2.67	–	–	–	–	–	–	2.00	0.63
35	*Cladosporium* sp. 3	–	–	–	–	–	–	1.00	1.33	1.00	0.32
36	*Cladosporium sphaerospermum*	–	–	–	–	–	–	1.00	1.33	1.00	0.32
37	*Cryptococcus* sp.	–	–	–	–	1.00	1.33	–	–	1.00	0.32
38	*Eupenicillium* cf. *javanicum*	–	–	–	–	1.00	1.33	–	–	1.00	0.32
39	*Geotrichum* sp.	–	–	1.00	1.33	–	–	–	–	1.00	0.32
40	*Gliomastix* sp.	–	–	1.00	1.33	–	–	–	–	1.00	0.32
41	*Hortaea wernickii*	2.00	2.67	–	–	–	–	1.00	1.33	3.00	0.95
42	Hyphomycete 1	–	–	1.00	1.33	–	–	–	–	1.00	0.32
43	Hyphomycete 2	1.00	1.33	–	–	–	–	–	–	1.00	0.32
44	Hyphomycete 3	–	–	–	–	–	–	1.00	1.33	1.00	0.32
45	Hyphomycete 4	–	–	1.00	1.33	–	–	–	–	1.00	0.32
46	Hyphomycete 5	1.00	1.33	2.00	2.67	1.00	1.33	–	–	4.00	1.27
47	Hyphomycete 6	–	–	–	–	–	–	1.00	1.33	1.00	0.32
48	Hyphomycete 7	–	–	1.00	1.33	–	–	–	–	1.00	0.32
49	Hyphomycete 8	–	–	–	–	–	–	1.00	1.33	1.00	0.32
50	Hyphomycete 9	3.00	4.00	3.00	4.00	2.00	2.67	3.00	4.00	11.00	3.48
51	Hyphomycete 10	–	–	2.00	2.67	–	–	–	–	2.00	0.63
52	Hyphomycete 11	1.00	1.33	–	–	–	–	–	–	1.00	0.32
53	*Kloeckera* sp.	–	–	–	–	2.00	2.67	–	–	2.00	0.63
54	*Mammaria* sp.	–	–	–	–	–	–	1.00	1.33	1.00	0.32
55	*Mycelia sterilia* 1	2.00	2.67	2.00	2.67	1.00	1.33	–	–	5.00	1.58
56	*Mycelia sterilia* 2	1.00	1.33	3.00	4.00	–	–	2.00	2.67	6.00	1.90
57	*Mycelia sterilia* 3	–	–	2.00	2.67	–	–	–	–	2.00	0.63
58	*Mycelia sterilia* 4	1.00	1.33	–	–	–	–			1.00	0.32
59	*Mycelia sterilia* 5	2.00	2.67	–	–	–	–	1.00	1.33	3.00	0.95
60	*Mycelia sterilia* 6	–	–	–	–	1.00	1.33	1.00	1.33	2.00	0.63
61	*Mycelia sterilia* 7	–	–	–	–	1.00	1.33	1.00	1.33	2.00	0.63
62	*Mycelia sterilia* 8	–	–	–	–	–	–	1.00	1.33	1.00	0.32
63	*Mycelia sterilia* 9	–	–	–	–	2.00	2.67	–	–	2.00	0.63
64	*Mycelia sterilia* 10	1.00	1.33	1.00	1.33	1.00	1.33	–	–	3.00	0.95
65	*Mycelia sterilia* 11	2.00	2.67	1.00	1.33	–	–	–	–	3.00	0.95
66	*Mycelia sterilia* 12	–	–	2.00	2.67	–	–	–	–	2.00	0.63
67	*Mycelia sterilia* 13	–	–	1.00	1.33	–	–	–	–	1.00	0.32
68	*Mycelia sterilia* 14	–	–	3.00	4.00	–	–	–	–	3.00	0.95
69	*Mycelia sterilia* 15	–	–	–	–	1.00	1.33	–	–	1.00	0.32
70	*Mycelia sterilia* 16	1.00	1.33	–	–	1.00	1.33	–	–	2.00	0.63
71	*Mycelia sterilia* 17	1.00	1.33	1.00	1.33	–	–	–	–	2.00	0.63
72	*Mycelia sterilia* 18	1.00	1.33	1.00	1.33	–	–	–	–	2.00	0.63
73	*Mycelia sterilia* 19	3.00	4.00	–	–	–	–	–	–	3.00	0.95
74	*Mycelia sterilia* 20	3.00	4.00	–	–	–	–	–	–	3.00	0.95
75	*Mycelia sterilia* 21	–	–	–	–	8.00	10.67	–	–	8.00	2.53
76	*Neosartorya* sp.	–	–	-	–	–	–	2.00	2.67	2.00	0.63
77	*Paecilomyces javanicus*	1.00	1.33	–	–	–	–	–	–	1.00	0.32
78	*Paecilomyces* cf. *lilacinus*	3.00	4.00	–	–	–	–	–	–	3.00	0.95
79	*Paecilomyces* cf. *persicinus*	1.00	1.33	11.00	14.67	–	–	–	–	12.00	3.80
80	*Paecilomyces* cf. *roseolus*	2.00	2.67	–	–	2.00	2.67	2.00	2.67	6.00	1.90
81	*Paecilomyces* sp. 1	1.00	1.33	–	–	–	–	–	–	1.00	0.32
82	*Paecilomyces* sp. 2	–	–	1.00	1.33	–	–	–	–	1.00	0.32
83	*Paecilomyces* sp. 3	1.00	1.33	–	–	–	–	–	–	1.00	0.32
84	*Paecilomyces* sp. 4	–	–	–	–	–	–	1.00	1.33	1.00	0.32
85	*Paecilomyces* sp. 5	–	–	1.00	1.33	–	–	–	–	1.00	0.32
86	*Paecilomyces victoriae*	3.00	4.00	–	–	1.00	1.33	–	–	4.00	1.27
87	*Penicillium chrysogenum*	2.00	2.67	2.00	2.67	2.00	2.67	5.00	6.67	11.00	3.48
88	*Penicillium* cf. *citreonigrum*	–	–	1.00	1.33	–	–	–	–	1.00	0.32
89	*Penicillium* cf. *citrinum*	–	–	1.00	1.33	–	–	1.00	1.33	2.00	0.63
90	*Penicillium* cf. *janthinellum*	7.00	9.33	–	–	–	–	3.00	4.00	10.00	3.16
91	*Penicillium purpurescens*	1.00	1.33	–	–	–	–	–	–	1.00	0.32
92	*Penicillium rubrum*	–	–	1.00	1.33	–	–	–	–	1.00	0.32
93	*Penicillium rugulosum*	–	–	1.00	1.33	–	–	–	–	1.00	0.32
94	*Penicillium* sp. 1	–	–	1.00	1.33	–	–	–	–	1.00	0.32
95	*Penicillium* sp. 2	–	–	2.00	2.67	–	–	–	–	2.00	0.63
96	*Penicillium* sp. 3	–	–	3.00	4.00	–	–	2.00	2.67	5.00	1.58
97	*Penicillium* sp. 4	–	–	1.00	1.33	–	–	–	–	1.00	0.32
98	*Penicillium* sp. 5 Sect. Aspergilloides	2.00	2.67	–	–	–	–	–	–	2.00	0.63
99	*Penicillium* sp. 6	2.00	2.67	–	–	–	–	2.00	2.67	4.00	1.27
100	*Penicillium spinulosum*	2.00	2.67	1.00	1.33	–	–	–	–	3.00	0.95
101	*Pestalotiopsis* sp.	2.00	2.67	–	–	–	–	–	–	2.00	0.63
102	*Pichia angusta*	–	–	–	–	1.00	1.33	–	–	1.00	0.32
103	*Ramichloridium* sp.	1.00	1.33	–	–	–	–	–	–	1.00	0.32
104	*Rhinocladiella* sp.	1.00	1.33	–	–	–	–	–	–	1.00	0.32
105	*Scedosporium aurantiacum*	1.00	1.33	–	–	–	–	–	–	1.00	0.32
106	*Stachybotrys* sp.	–	–	–	–	1.00	1.33	–	–	1.00	0.32
107	*Trichoderma aureoviride*	–	–	–	–	1.00	1.33	–	–	1.00	0.32
108	*Trichoderma* cf. *polysporum*	–	–	3.00	4.00	–	–	–	–	3.00	0.95
109	*Tritirachium oryzae*	–	–	–	–	–	–	4.00	5.33	4.00	1.27
110	Yeast 1	–	–	–	–	2.00	2.67	–	–	2.00	0.63
Summary											
Total no. of species		54		45		27		33			
Shannon index of diversity: H’		3.39		3.57		3.02		3.33			
Simpson index of diversity: D		0.06		0.03		0.05		0.02			
Simpson index of diversity: 1/D		15.52		36.59		20.7		40.14			
Shannon index of eveness: J’		0.85		0.94		0.92		0.95			
Simpson index of eveness: E_1/D_		0.29		0.81		0.77		1.22			

**Figure 2.**
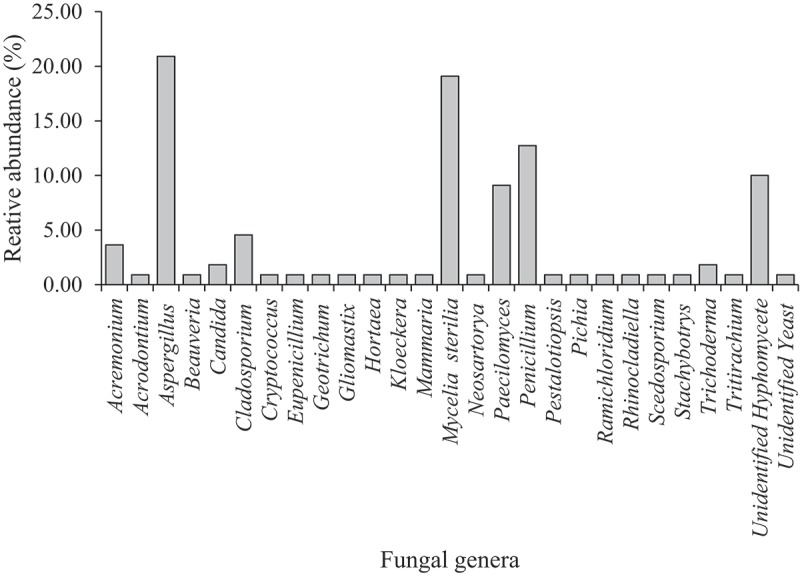
Relative abundance of sponge-associated fungal genera isolated in mangrove-attached sponges collected from New Washington, Aklan, Philippines.

Based on the percentage of total isolates of all mangrove-associated sponges, *Aspergillus* dominated the fungal genera under asexual morphs (*n *= 23, 20.91%) followed by *Mycelia sterilia* (*n *= 21, 16.28%), *Penicillium* (*n *= 14, 12.73%), *Paecilomyces* (*n *= 10, 9.09%) and unidentified hyphomycete (*n = *11, 8.53%) (Figure 2). In addition, genera under *Cladosporium* and *Acremonium* have 5 (4.55%) and 4 (3.64%) species, respectively. Furthermore, two species (1.82%) were isolated under the genera *Candida* and *Trichoderma* while only 1 species each (0.91%) were isolated under the genera *Acrodontium, Beauveria, Cryptococcus, Geotrichum, Gliomastix, Hortaea, Kloeckera, Mammaria, Pestalotiopsis, Pichia, Ramichloridium, Rhinocladiella, Scedosporium, Stachybotrys* and *Tritirachium*. Sexual morphs were represented by four genera, *Candida* (2 species, 1.82%) *Eupenicillium* (1 species, 0.91%), *Neosartorya* (1 species) and *Tritirchium* (1 species).

Based on the total frequency of occurrence (%), *Acrodontium* cf. *crateriforme* (11.39%) and *Aspergillus niger* (8.23%) were reported as very frequent and frequent species, respectively (Table 1). Seventeen species (*Aspergillus sydowii, Aspergillus* cf. *fumisynnematus, Aspergillus* sp. 11, Sect. Terreus, *Candida guilliermondii*, Hyphomycete 5, Hyphomycete 9, *Mycelia sterilia* 1, *Mycelia sterilia* 2, *Mycelia sterilia* 21, *Paecilomyces* cf. *persicinus, Paecilomyces* cf. *roseolus, Paecilomyces victoriae, Penicillium* cf. *janthinellum, Penicillium chrysogenum, Penicillium* sp. 3, *Penicillium* sp. 6, *Tritirachium oryzae*) were recorded as infrequent. Other 91 species were recorded as rare.

The mangrove sponge *Halichondria* cf. *panicea* harboured the most fungal isolates with 54 species, followed by *Axinella* sp. (45 isolates), *Haliclona* sp. (33 species) and *Tedania* sp. (27 species) (Table 1). *Axinella* sp. had the highest Shannon index of diversity value (*H*’: 3.57) while *Haliclona* sp. had the highest Shannon (*J*’) and Simpson (E_1/*D*_) index of evenness with a value of 0.95 and 1.22, respectively. *Halichondria* cf. *panicea* had the highest Simpson index of dominance (*d*’) with a value of 0.06.

The Jaccard’s coefficient of similarity (*J’*), based on the presence or absence of each fungus, was calculated among different sponge species to compare the composition of fungi on each sponge host (Table 2). Based on pair-wise comparison of similarities of 110 fungal isolates on four sponges, the similarity index was highest between *Halichondria* cf. *panicea* vs *Axinella* sp. (0.22) followed by both *Halichondria* cf. *panicea vs Tedania* sp. and *Halichondria* cf. *panicea* vs *Haliclona* sp. with the *J’* value of 0.19. It was least between *Axinella* sp. and *Haliclona* sp. with *J’* value of 0.13.

**Table 2.** Jaccard’s coefficient of similarity (J’) for fungal species among sponge species.

	*Axinella* sp.	*Halichondria* cf. *panicea*	*Haliclona* sp.
*Halichondria* cf. *panicea*	0.22		
*Haliclona* sp.	0.13	0.19	
*Tedania* sp.	0.14	0.19	0.18

### Association of fungi on the different mangrove-attached sponges

Three classifications were done based on the presence of fungal genera in certain number of sponge species, as adapted from Li and Wang (2009). “Sponge-generalists” are genera that can be found in all sponge species and results showed that, the genera *Acrodontium, Aspergillus, Candida, Paecilomyces* and *Penicillium* could be classified under this group. The genera *Acremonium, Cladosporium, Hortaea* and *Trichoderma* are classified as sponge-associates since they were identified on more than one sponge. The “sponge-specialists” would include the genera *Beauveria, Cryptococcus, EuPenicillium, Geotrichum, Gliomastix, Kloeckera, Mammaria, Neosartorya, Pestalotiopsis, Pichia, Ramichloridium, Rhinocladiella, Scedosporium, Stachybotrys* and *Tritirachium*.

### Fungal load of mangrove-associated sponges

The total fungal load of the four species of mangrove-associated sponges yielded varying counts on the various culture media used (Table 3). For *Halichondria* cf. *panicea*, the highest fungal density was recorded from cornmeal agar and lowest value from potato dextrose agar. The highest CFU in *Axinella* sp. was recorded from CMA and lowest fungal load from MBA. MBA had the highest fungal load in *Tedania* sp. and lowest value from CMA. For *Haliclona* sp., RBACl had the highest fungal load value and lowest CFU from MBA.

**Table 3.** Fungal density (CFU g-1) of different mangrove-associated sponges in five culture media.

Agar	*Axinella* sp.	*Halichondria* cf. *panacea*	*Haliclona* sp.	*Tedania* sp.
PDA	1.47 × 10^2^	7.67 × 10^1^	2.23 × 10^2^	2.00 × 10^2^
MBA	6.00 × 10^1^	2.07 × 10^2^	1.50 × 10^2^	3.10 × 10^2^
CDA	1.20 × 10^2^	1.37 × 10^2^	3.27 × 10^2^	2.67 × 10^2^
RBACl	1.83 × 10^2^	3.17 × 10^2^	3.67 × 10^2^	2.37 × 10^2^
CMA	3.43 × 10^2^	7.13 × 10^2^	2.20 × 10^2^	1.10 × 10^2^

PDA- Potato Dextrose Agar; MBA- Mycobiotic Agar; CDA- Czapek Dox Agar; RBACl- Rose Bengal Agar with chloramphenicol; CMA- Cornmeal Agar.

*Halichondria* cf. *panicea* had the highest fungal load value (7.13 × 10^2^) while the lowest CFU/g was recorded from *Axinella* sp. (6.00 × 10^1^)

## Discussion

### Fungal diversity in mangrove-associated sponges

The study on the ecological role, including its diversity and association, of fungi on marine sponges are still scarce and data were largely generated due to the diversity of novel bioactive metabolites produced with promising biotechnological applications (Höller et al. 2000; Bugni and Ireland 2004; Konig et al. 2006; Wang 2006; Raghukumar 2008; Aly et al. 2011; Debbab et al. 2011; Jones 2011). Fungi were mostly isolated from subtidal sponges but there is no published information on the fungal associates of mangrove-associated sponges. To date, only bacterial communities from mangroves sponges collected from the Caribbean Sea were reported (Yang et al. 2011). Information on the role of fungi in mangrove sponges has not yet been studied but some mycologists suggest that the role of fungi in sponges include nutrient transfer and chemical defence (Bugni and Ireland 2004; Taylor et al. 2007; Ding et al. 2011). On the other hand, sponge-associated bacteria enhance the endemism of this invertebrate by degrading mangrove-derived DOM and other organic compounds which is important in organic matter assimilation leading to the survival of mangrove species and the exclusion of typical reef species (Hunting et al. 2010, 2013).

The present study gives an insight on the diversity of fungi associated with mangrove sponges. The fungal communities of mangrove sponges were composed mainly of asexual morphs with hyphomycetes represented by 87 species. Previous studies also observed asexual morphs dominating the fungal assemblages of subtidal sponges (Wang et al. 2008; Ding et al. 2011; Thirunavukkarasu 2012). The genera *Aspergillus* (21.52%), *Penicillium* (14.24%) and *Acrodontium* (11.39%) were recorded as very frequent while *Paecilomyces* (9.81%) was a frequently occurring genus (Table 1). Infrequently occurring genera include *Cladosporium* (3.16%), *Acremonium* (2.53%), *Trichoderma* (2.53%) and *Candida* (1.90%). Other genera were recorded as rare. It includes *Beauveria* and *Tritirachium* where previous works (Höller et al. 2000; Paz et al. 2010; Wiese et al. 2011) considered it also to be isolated rarely from marine sponges.

These mangrove sponges were collected in the same location but the composition of fungal genera differs from one another except *Acrodontium, Aspergillus, Candida, Paecilomyces* and *Penicillium* that were isolated in all sponge species. The genera *Beauveria, Cryptococcus, Eupenicillium, Kloeckera, Pichia, Stachybotrys* were only isolated in *Tedania* sp.; *Geotrichum* and *Gliomastix* in *Axinella* sp.; *Mammaria, Neosartorya, Tritirachium* in *Haliclona* sp.; *Pestalotiopsis, Ramichloridium, Rhinocladiella, Scedosporium* in *Halichondria* cf. *panicea*. The differences in the fungal composition on the different sponge species suggest host-preference of the different fungal taxa. Furthermore, the results in this study and previous works in subtidal sponges (Höller et al. 2000; Wang 2006; Wang et al. 2008; Liu et al. 2010; Yu et al. 2012) showed that the differences in the fungal composition and its diversity may be attributed to the species of sponges with various morphological structures. Ding et al. (2011), on his work on South China Sea sponges (*Clathrina luteoculcitella* and *Holoxea* sp.) sampled in the same location, also observed this wherein orders *Agaricales, Boliniales, Microascales, Mucorales, Pleosporales, Saccharomycetales* and *Xylariales* were only isolated from sponge *Clathrina luteoculcitella* but not in *Holoxea* sp. Thus, even the sponges were collected on the same location, they harbour different isolates which suggested that these isolates were not spores from seawater column and trapped during the filter feeding process of sponges. Previous studies by Gao et al. (2008), Li and Wang (2009) and Jin et al. (2014) demonstrated that fungal communities isolated from sponges differ from the surrounding water. For example, *Penicillium janthinellum, Fusarium solani* and *P. chrysogenum* which were isolated from seawater samples but not present within sponges (Li and Wang 2009). However, it is insufficient to disprove sponge-specific nature of a microbe by merely proving the presence of microbe outside a sponge. A predator or storm for example may damage sponge and microbes associated with it may disintegrate and spread into the seawater column (Taylor et al. 2007).

Diversity of fungi associated with marine sponges remains an understudied area and more evidence is required to elucidate their possible ecological role (Gao et al. 2008; Wang et al. 2008). The present investigation does not show any direct evidence that the isolated fungi have been actively growing on the sponge tissues. As a result, the difficulty also arises on how to determine whether they are sponge-symbiotic fungi or not. So far, there is little evidence regarding the symbiotic relationship between sponges and fungi. For example, Maldonado et al. (2005) showed direct evidence of sponge-endosymbiotic yeasts in a marine sponge *Chondrilla* sp. that is transmitted maternally through fertilised eggs based using immunocytochemical technique to label the β-1,4-N-acetyl-D-glucosamine residues of chitin walls. There is also indirect evidence of the putative fungal original intron in a sponge *Tetilla* sp., as observed by Rot et al. (2006), perhaps because of horizontal gene transfer. In addition, marine ascomycetes of the genus *Koralionastes* have been reported to be in some way associated with crustaceous sponges wherein it forms fruiting bodies only in close association with the sponges associated with corals (Kohlmeyer and Volkmann-Kohlmeyer 1990). Furthermore, Perovic-Ottstadt et al. (2004) demonstrated the presence of receptor proteins for fungal cell wall components (e.g. (1→3)-β-d-glucan-binding proteins), in the marine sponge *Suberetis domuncula, which* indicates that sponges are biochemically equipped for dealing with fungi. Several bacteria and archaea, along with the ubiquitous fungus *Penicillium*, as reported by Simister et al. (2012), are symbionts of sponges. Using immunocytochemistry, transmission electron microscopy (TEM) technique and non-cultivation-dependent analysis, the real association between fungi and marine sponges will be confirmed including vital role or functions of fungi in the sponge (Maldonado et al. 2005; Passarini et al. 2013). Höller et al. (2000) proposed that investigation of a larger number of samples and surrounding water should be done to determine if sponge-associated fungi are not terrestrial fungi filtered from the surrounding waters but adapted to the marine habitat and on its host.

Even employing diverse culture media, 21 species remained sterile. Höller et al. (2000) also isolated 37 strains of *Mycelia sterilia* in 14 sponges even using diverse culture media and culture conditions to induce sporulation of fungi. Molecular analysis can be of great help to identify fungi with no reproductive structures (e.g. conidia and ascomata) Furthermore, 11 species of hyphomycetes remained unidentified and requires further investigation including molecular analysis for identification.

### Comparison of culturable fungal diversity of sponges

In this study, the genera *Acrodontium, Aspergillus, Candida, Paecilomyces* and *Penicillium* were considered sponge-generalists while *Acremonium, Cladosporium, Hortaea* and *Trichoderma* are classified as sponge-associates. The sponge-specialists include the genera *Beauveria, Cryptococcus, Eupenicillium, Geotrichum, Gliomastix, Kloeckera, Mammaria, Neosartorya, Pestalotiopsis, Pichia, Ramichloridium, Rhinocladiella, Scedosporium, Stachybotrys* and *Tritirachium. Aspergillus, Penicillium* and *Eupenicillium* were considered by Li and Wang (2009) as “sponge-generalists” but *Eupenicillium* was “sponge-associate” in this study. *Candida*, on the other hand, was “sponge-generalist” but it was “sponge-specialist” on the study of Li and Wang (2009). *Trichoderma* was considered “sponge-associate” in this study and this is in line with result of Höller et al. (2000) but Menezes et al. (2010) classified this as “sponge-generalist”. Table 4 shows the summary of fungal genera associated with marine sponges recovered from various locations. The genera *Cladosporium* (39 sponge species), *Aspergillus* (47 sponge species), *Penicillium* (53 sponge species), *Acremonium* (41 sponge species) and *Trichoderma* (29 sponge species) were common marine fungi recovered from the marine sponges and can be considered sponge-generalists (Höller et al. 2000; Morrison-Gardiner 2002; Gesner et al. 2005; Proksch et al. 2008; Wang et al. 2008; Ein-Gil et al. 2009; Li and Wang 2009; Liu et al. 2010; Paz et al. 2010; Ding et al. 2011; Thirunavukkarasu et al. 2012).

**Table 4.** Fungal genera associated with sponges recovered in different locations (genus level).

Fungus	Sponge	Reference
Ascomycetes		
*Acremonium*	*Amphilectus digitata; Amphimedon viridis; Aplysina aerophoba; Aplysina cauliformis; Aplysina fulva; Axinella* sp. 1; *Axinella* sp. 2; *Biemna fistulosa; Callyspongia* sp. cf.*C. flammea; Callyspongia vaginalis; Cliona viridis; Cynachirella alloclada; Desmapsamma anchorata; Dragmacidon reticulatum; Ectyoplasia perox; Halichondria panicea; Haliclona caerulea; Homaxinella subdola; Hyattella cribriformis; Hyatella* cf. *intestinalis; Ircinia variabilis; Leucosolenia* sp.; *Lissodendoryx colombiensis; Myxilla incrustans; Neofibularia nolitangere; Niphates erecta; Oscarella lobularis; Placospongia intermedia; Petrosia ficiformis; Psammocinia* sp.; *Raspailia ramosa; Scopalina* sp.; *Sigmadocia pumila; Spirastrella* sp.; *Suberites carnosus; Suberites domuncula; Sycon* sp. *Tethya aurantium; Xestospongia* sp.; *Halichondria* cf. *panicea; Haliclona* sp.	Höller et al. (2000), Pivkin et al. (2006), Proksch et al. (2008), Menezes et al. (2010), Paz et al. (2010), Wiese et al. (2011), Thirunavukkarasu et al. (2012), Vasanthabharathi and Jayalakshmi, (2012), Flemer (2013), and Bolaños et al. (2015); Present study
*Acrodontium*	*Axinella* sp.; *Halichondria* cf. *panicea; Tedania* sp.; *Haliclona* sp.	Present study
*Aspergillus*	*Amphilectus digitata; Amphimedon viridis; Aplysinopsis* sp.; *Biemna fistulosa; Callyspongia diffusa; Callyspongia* sp.; *Clathrina luteoculcitella; Cliona celata; Cliona quadrata; Cliona viridis; Dragmacidon reticulatum; Fasciospongia cavernosa; Gelliodes carnosa; Gelliodes fibrosa; Halichondria panicea; Haliclona caerulea; Haliclona madrepora; Haliclona oculata; Haliclona simulans; Hyattella cribriformis; Hymeniacidon assimilis; Holoxea* sp.; *Homaxinella subdola; Ircinia oros; Ircinia variabilis; Lissodendoryx sinensis; Mycale armata; Mycale fibrexilis; Mycale laxissima; Petrosia ficiformis; Phakettia cribrosa; Psammocinia* sp.; *Pseudosuberites andrewi; Sigmadocia carnosa; Sigmadocia pumila; Spongia officinalis var. ceylonensis; Suberites carnosus; Suberites domuncula; Suberites zeteki; Tedania ignis; Tethya aurantium; Theonella swinhoei; Xestospongia testudinaria; Axinella* sp.; *Halichondria* cf. *panicea; Tedania* sp.; *Haliclona* sp.	Höller et al. (2000), Pivkin et al. (2006), Gao et al. (2008), Proksch et al. (2008), Wang et al. (2008), Baker et al. (2009), Li and Wang (2009), Liu et al. (2010), Menezes et al. (2010), Paz et al. (2010), Ding et al. (2011), Wiese et al. (2011), Zhou et al. (2011), Meenupriya and Thangaraj (2010), Thirunavukkarasu et al. (2012), Vasanthabharathi and Jayalakshmi (2012), Flemer (2013), Jin et al. (2014), and Bolaños et al. (2015); Present study
*Beauveria*	*Amphimedon compressa; Aplysina fulva; Aplysina* sp.; *Ectyoplasia perox; Halichondria panacea; Myxilla incrustans; Niphates erecta; Suberites zeteki; Tethya aurantium; Tedania* sp.	Höller et al. (2000), Wang et al. (2008), Wiese et al. (2011), and Bolaños et al. (2015); Present study
*Candida*	*Amphilectus fucorum; Clathrina luteoculcitella; Cliona celata; Haliclona simulans; Leucosolenia* sp.; *Mycale armata; Suberites carnosus; Axinella* sp.; *Halichondria* cf. *panicea; Tedania* sp.; *Haliclona* sp.	Li and Wang (2009), Liu et al. (2010), Ding et al. (2011), and Flemer (2013); Present study
*Cladosporium*	*Amphimedon viridis; Amphilectus digitata; Amphilectus fucorum; Aplysina aerophoba; Aplysina chiriquensis; Axinella dissimilis; Axinella* sp. *1; Callyspongia vaginalis; Chondrilla* sp.; *Clathrina luteoculcitella; Cliona viridis; Dragmacidon reticulatum; Ectyoplasia perox; Gelliodes carnosa; Gelliodes fibrosa; Halichondria panicea; Haliclona caerulea; Haliclona simulans; Hyatella cf intestinalis; Ircinia oros; Leucosolenia* sp.; *Lissodendoryx colombiensis; Mycale armata; Mycale laxissima; Myxilla incrustans; Oscarella lobularis; Psammocinia* sp.; *Pseudosuberites andrewi; Raspailia ramosa; Spirastrella* sp.; *Suberites domuncula; Suberites zeteki; Sycon* sp.; *Tethya aurantium; Theonella swinhoei; Xestospongia testudinaria; Axinella* sp.; *Halichondria* cf. *panicea; Haliclona* sp.	Höller et al. (2000), Pivkin et al. (2006), Gao et al. (2008), Proksch et al. (2008), Li and Wang (2009), Liu et al. (2010), Menezes et al. (2010), Paz et al. (2010), Ding et al. (2011), Wiese et al. (2011), Thirunavukkarasu et al. (2012), Flemer (2013), Passarini et al. (2013), Jin et al. (2014), and Bolaños et al. (2015); Present study
*Eupenicillium*	*Callyspongia* sp. cf. *C. flammea; Dragmacidon reticulatum; Gelliodes fibrosa; Gelliodes fibrosa; Halichondria panicea; Haliclona caerulea; Hymeniacidon assimilis; Mycale armata; Psammocinia* sp.; *Sycon* sp.; *Tedania* sp.	Höller et al. (2000), Pivkin et al. (2006), Li and Wang (2009), Paz et al. (2010), Passarini et al. (2013); Present study
*Geotrichum*	*Sycon* sp.; *Axinella* sp.	Höller et al. (2000); Present study
*Gliomastix*	*Psammocinia* sp.; *Suberites domuncula; Axinella* sp.	Proksch et al. (2008) and Paz et al. (2010) Present study
*Hortaea*	*Gelliodes carnosa; Haliclona simulans; Mycale armata; Halichondria* cf. *panicea; Haliclona* sp.	Gao et al. (2008) and Liu et al. (2010); Present study
*Kloeckera*	*Tedania* sp.	Present study
*Mammaria*	*Haliclona* sp.	Present study
*Neosartorya* sp.	*Aka coralliphaga; Chondrilla australiensis; Haliclona* sp.	Eamvijarn et al. (2013) and Gomes et al. (2013); Present study
*Paecilomyces*	*Clathrina luteoculcitella; Dragmacidon reticulatum; Ectyoplasia perox; Halichondria panacea; Haliclona simulans; Holoxea* sp.; *Leucosolenia* sp.; *Myxilla incrustans; Oscarella lobularis; Petrosia ficiformis; Suberites domuncula; Tethya aurantium; Axinella* sp.; *Halichondria* cf. *panicea; Haliclona* sp.; *Tedania* sp.	Höller et al. (2000), Baker et al. (2009), Proksch et al. (2008), Ding et al. (2011), Wiese et al. (2011), and Passarini et al. (2013); Present study
*Penicillium*	*Agelas clathrodes; Amphilectus digitata; Amphilectus fucorum; Amphimedon viridis; Aplysina aerophoba; Callyspongia diffusa; Callyspongia* sp. cf.*C. flammea; Callyspongia vaginalis; Clathrina luteoculcitella; Cliona celata; Cliona quadrata; Cliona viridis; Dragmacidon reticulatum; Ectyoplasia perox; Gelliodes carnosa; Gelliodes fibrosa; Halichondria panicea; Haliclona caerulea; Haliclona simulans; Holoxea* sp.; *Homaxinella subdola; Hyattella cribriformis; Hymeniacidon assimilis; Ircinia oros; Ircinia variabilis; Leucosolenia* sp.; *Lissodendoryx sinensis; Mycale armata; Mycale fibrexilis; Mycale laxissima; Myxilla incrustans; Pericharax heteroraphis; Petrosia ficiformis; Phakettia cribrosa; Polymastia boletiformis; Psammocinia* sp.; *Pseudosuberites andrewi; Raspailia ramosa; Rhizaxinella* sp.; *Sigmadocia pumila; Spirastrella* sp.; *Spongia officinalis var. ceylonensis; Stelligera stuposa; Suberites ficus; Suberites carnosus; Suberites domuncula; Suberites zeteki; Sycon* sp.; *Tethya aurantium; Axinella* sp.; *Halichondria* cf. *panicea; Haliclona* sp.; *Tedania* sp.	Höller et al. (2000), Pivkin et al. (2006), Gao et al. (2008), Proksch et al. (2008), Wang et al. (2008), Baker et al. (2009), Li and Wang (2009), Liu et al. (2010), Menezes et al. (2010), Paz et al. (2010), Ding et al. (2011), Wiese et al. (2011), Zhou et al. (2011), and Thirunavukkarasu et al. (2012), Vasanthabharathi and Jayalakshmi, (2012), Flemer (2013), Passarini et al. (2013), Bolaños et al. (2015); Present study
*Pestalotiopsis*	*Aplysina cauliformis; Aplysina fulva; Aplysina* sp. *6; Clathrina luteoculcitella; Dragmacidon reticulatum; Mycale* sp.; *Halichondria* cf. *panicea*	Caballero-George et al. (2010), Ding et al. (2011), Passarini et al. (2013), and Bolaños et al. (2015); Present study
*Pichia*	*Tedania* sp.	Present study
*Ramichloridium*	*Halichondria* cf. *panicea*	Present study
*Rhinocladiella*	*Halichondria* cf. *panicea*	Present study
*Scedosporium*	*Halichondria* cf. *panicea*	Present study
*Stachybotrys*	*Ircinia oros; Psammocinia* sp.; *Sycon* sp.; *Tedania* sp.	Höller et al. (2000) and Paz et al. (2010); Present study
*Trichoderma*	*Amphimedon viridis; Aplysina* sp.; *Biemna fistulosa; Callyspongia diffusa; Callyspongia* sp.; *Callyspongia vaginalis; Cliona viridis; Dragmacidon reticulatum; Ectyoplasia perox; Gelliodes fibrosa; Halichondria panicea; Haliclona madrepora; Haliclona simulans; Hyattella cribriformis; Hymeniacidon assimilis; Mycale fibrexilis; Mycale laxissima; Mycale* sp.; *Myxilla incrustans; Niphates erecta; Psammocinia* sp.; *Sigmadocia pumila; Spongia officinalis var. ceylonensis; Suberites domuncula; Suberites zeteki; Sycon* sp.; *Tethya aurantium; Axinella* sp.; *Tedania* sp.	Höller et al. (2000), Pivkin et al. (2006), Proksch et al. (2008), Wang et al. (2008); Baker et al. (2009b), Li and Wang (2009), Caballero-George et al. (2010), Meenupriya and Thangaraj (2010), Menezes et al. (2010), Paz et al. (2010), Wiese et al. (2011), Zhou et al. (2011), Thirunavukkarasu et al. (2012), Vasanthabharathi and Jayalakshmi, (2012), and Passarini et al. (2013); Present study
Basidiomycetes		
*Cryptococcus*	*Gelliodes carnosa; Tedania* sp.	Liu et al. (2010); Present study
*Tritirachium*	*Pseudosuberites andrewi; Haliclona* sp.	Thirunavukkarasu et al. (2012); Present study
Unidentified hyphomycete	*Axinella* sp.; *Halichondria* cf. *panicea; Haliclona* sp.; *Tedania* sp.	Present study
Unidentified yeast sp.	*Tedania* sp.	Present study
*Mycelia sterilia*	*Callyspongia diffusa; Callyspongia* sp. cf.*C. flammea; Callyspongia vaginalis; Cliona quadrata; Cliona viridis; Ectyoplasia perox; Haliclona madrepora; Halichondria panacea; Homaxinella subdola; Hymeniacidon assimilis; Lissodendoryx sinensis; Leucosolenia* sp.; *Neofibularia nolitangere; Phakettia cribrosa; Pseudosuberites andrewi; Sigmadocia pumila; Suberites carnosus; Suberites domuncula; Sycon* sp.; *Axinella* sp.; *Halichondria* cf. *panicea; Tedania* sp.; *Haliclona* sp.	Höller et al. (2000), Pivkin et al. (2006), Proksch et al. (2008), and Thirunavukkarasu et al. (2012); Present study

It is difficult to suggest that the present study isolated sponge-specialist based on the differences of fungi recovered from four mangrove sponges because there is no direct evidence and there are limited studies on fungal associates of mangrove sponges. Extensive survey of fungi in more species of sponges including comparison on the same species in this study but different geographical locations and using biochemical and molecular methods (e.g. 454 pyrosequencing) could reveal the sponge-fungal association.

In addition, the isolated fungal genera are common to terrestrial habitats, suggesting that these isolates may also be of terrestrial origin and can be considered, based on the definition of Kohlmeyer and Kohlmeyer (1979), facultative marine fungi but on the latest definition of Pang et al. (2016), these isolates were considered marine fungi. Marine fungi, as defined by Pang et al. (2016), are fungi that are recovered repeatedly from marine habitats because: (1) it can grow and/or sporulate (on substrata) in marine environments; (2) it forms symbiotic relationships with other marine organisms or (3) it is shown to adapt and evolve at the genetic level or be metabolically active in marine environments. If we based on the list of marine fungi by Jones et al. (2015), 16 species were considered marine fungi that include 9 species of *Aspergillus* (*A. candidus, A. niger, A. ochraceus, A*. cf. *penicilloides, A. restrictus, A. sclerotiorum, A. sydowii, A. tamarii, A. terreus*), 3 species of *Penicillium* (*P*. cf. *citreonigrum, P*. cf. *citrinum, P. spinulosum*), 1 *Trichoderma* species (*T. aureoviride*), 1 species of *Candida* (*C. guilliermondii*) and 2 species of *Cladosporium* (*C. cladosporioides, C. sphaerospermum*).

### Environment-dependent fungal diversity

The results of the present work and earlier studies show that the diversity sponge-associated fungi are more dependent on the surrounding environment where the sponge species thrives. For instance, no ascomycetes were isolated in mangrove sponge *Halichondria* cf. *panicea* collected from Aklan, Philippines while previous works of Höller et al. (2000) in Helgoland, Germany and Pivkin et al. (2006) in Sakhalin Island, Russia recovered ascomycetes in the subtidal sponge *Halichondria panicea*. Both present work and other published studies on *Halichondria* harbours *Acremonium, Aspergillus, Penicillium, Mycelia sterilia* and *Trichoderma*. Only Höller et al. (2000) isolated *Mucor*, a zygomycete. Furthermore, Flemer (2013) and Bolaños et al. (2015) recovered ascomycetes in subtidal sponges under the genus *Axinella* while no ascomycetes were isolated from mangrove sponge *Axinella* sp. collected in Aklan, Philippines. Only *Cladosporium* was isolated in the present work and two former studies in *Axinella dissimilis* (Flemer 2013) and *Axinella* sp. 1 (Bolaños et al. 2015). There is no similar species from the present work were recovered in *Axinella* sp. 2 and *Axinella* sp. 3 (Bolaños et al. 2015). Furthermore, between the sponge *Haliclona simulans*, collected from the coastal waters of Ireland (Baker et al. 2009), and Hainan Province of China (Liu et al. 2010). The fungal diversity between two different regions is quite different. For instance, the orders Capnodiales, Dothideales, Agaricostilbales, Wallemiales, which were present in the “Hainan” sample are not found in the “Irish” sample. In the “Irish” sample, fungi under the orders Chaetosphaeriales, Chaetothyrailes, Helotiales, Mucorales and Agaricomycotina, were isolated but were absent in the “Hainan” sample. Furthermore, no shared identical fungal species were observed in the two collections. The results of the comparison of the abovementioned studies supports the notion that being filter feeders, sponges enrich various fungal species from the surrounding seawater that are merely washed into the sea from their terrestrial habitats and just happen to survive in their “host organisms” (Höller et al. 2000; Taylor et al. 2007; Proksch et al. 2008; Wang et al. 2008; Liu et al. 2010; Wiese et al. 2011; Zhou et al. 2011). These remain dormant until plated onto a suitable culture medium (Wang et al. 2008). If such has been the case, the metabolic activities of fungi from sponges should be the same as that of those in other terrestrial environments. Surprisingly, these facultative marine fungi produce novel compounds that are different and not produced from their terrestrial conspecifics (Proksch et al. 2003; Konig et al. 2006; Wang 2006). Secondary metabolites in producing fungi play an important role in ecological interactions with other organisms allowing it to survive in its ecological niche while on its host, it enhance the defence mechanisms against pathogens and predators (Fox and Howlett 2008; Thomas et al. 2010).

## Conclusion

The fungal composition differs in each species of mangrove sponges even though they were collected in the same location suggesting that the isolates recovered were not merely seawater contamination and suggest sponge-preference by various fungal taxa that can be classified as true marine fungi. The development of marine fungi on these hosts appeared to be strongly influenced by the characteristics or nature of immediate environment.
